# Therapy-Associated Saliva and Taste change Evaluation (TASTE) in head & neck cancer patients undergoing radiotherapy: a study protocol

**DOI:** 10.1186/s12885-024-12497-y

**Published:** 2024-07-18

**Authors:** Anja Dietze, Peter J. Neyer, Marlene M. Speth, Philipp Metzler, Olgun Elicin, Panagiotis Balermpas, Daniel M. Aebersold, Oliver Riesterer, Sonja Stieb

**Affiliations:** 1 Center for Radiation Oncology KSA-KSB, Cantonal Hospital Aarau, Aarau, Switzerland; 2 Institute of Laboratory Medicine, Cantonal Hospital Aarau, Aarau, Switzerland; 3 Department of Otorhinolaryngology, Cantonal Hospital Aarau, Aarau, Switzerland; 4 Department of Oral- and Maxillofacial Surgery, Cantonal Hospital Aarau, Aarau, Switzerland; 5 Department of Radiation Oncology, Bern University Hospital and University of Bern, Inselspital, Bern, Switzerland; 6 Department of Radiation Oncology, University Hospital Zurich, University of Zurich, Zurich, Switzerland; 7 University of Zurich, Zurich, Switzerland

**Keywords:** Head and neck cancer, Radiotherapy, Taste impairment, Taste qualities, Xerostomia, Saliva, Quality of life

## Abstract

**Background:**

One of the main side effects of radiation therapy to the head and neck region is altered taste sensation. This causes significant morbidity and has profound effects on the quality of life (QoL) of patients. While radiation-associated toxicities like xerostomia and dysphagia are part of large investigations, data on taste impairment is sparse. Small cohort sizes in the majority of studies and a variety of analysis methods limit our current understanding of the underlying processes. None of the studies published to date used a taste-specific QoL questionnaire with differentiation of the different taste qualities (e.g. sour, bitter). Furthermore, data regarding the correlation of taste impairment with radiation-associated change in saliva composition is currently not available. The aim of the TASTE study is to fill this gap. Based on the acquired data, a normal tissue complication probability (NTCP) model for late radiation-associated taste impairment will be developed.

**Methods:**

In this prospective, observational multicenter study 150 head and neck cancer patients undergoing radiation therapy will be recruited and undergo repetitive (semi-) objective and subjective assessment of their taste, smell and salivary function (questionnaires, taste and smell assessment, saliva analysis). Primary endpoint will be patient-reported taste impairment 12 months post radiation therapy using a standardized questionnaire. Secondary endpoints will include taste impairment measured using taste strips at 12 months and 2 years post radiation therapy. Differences between subgroups (radiation side, chemotherapy, etc.) and changes over time will be assessed while adjusting for confounding factors (e.g. age, sex, smoking history).

**Discussion:**

This study sets out to further our understanding of taste impairment in patients undergoing radiation therapy to the head and neck region with the goal to prevent this common side effect in future patients. The results of the study may be used to evaluate taste-preserving radiotherapy for patients with head and neck cancer, which may significantly reduce the long-term burden in this patient cohort.

## Background

Radiation therapy is a cornerstone in the treatment of head and neck cancer patients. However, taste impairment is a frequent side effect of this treatment with major consequences on the patient’s quality of life (QoL). Over 90% of head and neck cancer patients undergoing radiotherapy experience moderate to severe taste disturbances in the acute phase [1]. This causes significant morbidity and distress, as altered taste perception is associated with reduced appetite, decreased food intake, and weight loss [2]. Patients that report severe taste impairment experience greater weight loss compared to patients that are less affected by this side effect [3]. One year post-treatment, altered sense of taste was reported among the most significant QoL issues by head and neck cancer patients [4]. Despite this, data on taste impairment post-radiation therapy is surprisingly sparse compared to other radiation-associated toxicities.

The sense of taste is a complex entity involving various tissues and intersecting with other senses (i.e. smell). It is mediated via taste receptor cells located in the taste buds. Taste buds are located primarily on the dorsum of the tongue but are also found on the surface of the oral cavity, oropharynx and upper third of the esophagus [5]. Hence, they are vulnerable to radiation damage during radiotherapy to the head and neck region. According to the receptor type most strongly activated, five taste modalities can be distinguished: sweet, sour, salty, bitter, and umami. Taste dysfunction can include a decreased sense of taste (hypogeusia), complete loss of taste (ageusia) or altered taste sensation (dysgeusia) [2, 5]. The pathomechanism of radiation-induced taste disturbances is not fully understood to date but might involve one or more of the following: direct and indirect damage to the taste buds or receptors, inflammation of the neural tissues transmitting signals, changes in the salivary flow and/or composition, loss, or distortions of olfactory function, as well as changes in the oral flora [6, 7].

The increasing patient-reported taste impairment over the course of radiotherapy indicates a dose/toxicity relationship. However, data on the impact of the radiation dose on taste impairment is limited and conflicting. Taste impairment has been correlated with the prescribed dose to the tumor [8], to the whole tongue [9, 10] or to the entire oral cavity [11–14]. Radiation can cause apoptosis in taste receptor cells and inhibit taste progenitor cell proliferation [6, 7]. Furthermore, a sharp decline in the number of taste buds has been observed after radiation, which might contribute to taste impairment as taste sensitivity is dependent on the size of the receptor field stimulated [10, 15]. It has been demonstrated that the dose to the taste bud bearing tongue mucosa differs significantly from the dose to the whole tongue [16]. Hence, it is conceivable that using taste-specific structures for dosimetric analysis of radiation-associated taste impairment rather than the whole tongue or entire oral cavity would be important. The only study to date using the taste bud bearing tongue mucosa as organ at risk (OAR) found no significant correlation of dose to this structure with taste impairment [17]. However, the lack of baseline taste values and follow up at regular time intervals present significant limitations of this study.

Another major contributor to taste disturbance that has been postulated is reduced salivary flow due to damage of the salivary glands during radiotherapy. Saliva is produced by major (parotid, submandibular, and sublingual) and minor salivary glands in the submucosa of the oral cavity and pharynx that are highly susceptible to radiation damage [18]. Xerostomia is a nearly ubiquitous side effect in the acute phase of radiotherapy in head and neck cancer patients and up to 40% of patients report long-term moderate to severe xerostomia even with modern radiotherapy techniques [19]. Food particles need to be in solution to stimulate taste receptor cells and saliva acting as a solvent permits their delivery to the taste buds [7]. Thus, reduced salivary volume and flow may impair physical contact of food with taste papillae. Salivary water, electrolytes and mucin have been shown to modulate taste sensitivity [20]. A correlation between xerostomia and taste impairment in patients undergoing radiotherapy of the head and neck region has been demonstrated previously [11–13, 17]. The relationship appears to be complex though, as primarily patient-reported xerostomia rather than reduced salivary output, was significantly correlated with severe taste dysfunction [11]. Overall, the precise role of radiation-induced xerostomia in decreased taste sensation remains uncertain to date.

### Objectives

Addressing the current lack of reliable data, the TASTE study aims to provide more detailed information on taste impairment in patients undergoing radiation therapy to the head and neck region. The objectives are as follows:

1. Primary objective: To develop a normal tissue complication probability (NTCP) model for late radiation-associated taste impairment, taking into account dose-volume histogram (DVH) data of the taste bud bearing tongue mucosa and salivary glands, baseline taste status, and baseline salivary gland function.
2. Secondary objectives:

- To longitudinally investigate changes in taste and smell associated with radiation therapy using standardized taste strips, sniffing sticks and QoL questionnaires.
- To evaluate the effects of the radiation dose to the taste bud bearing tongue mucosa on different taste qualities.
- To investigate the correlation between radiation dose to the salivary glands and saliva production/composition, and its impact on taste impairment.
- To analyze if radiation-associated taste impairment mid- or end of treatment predicts for late taste impairment.
- To define dose constraints for the taste bud bearing tongue mucosa and salivary glands with the goal of designing a study to evaluate a taste-preserving radiotherapy in head and neck cancer patients.

## Methods/design

### Study design

**Fig. 1:**
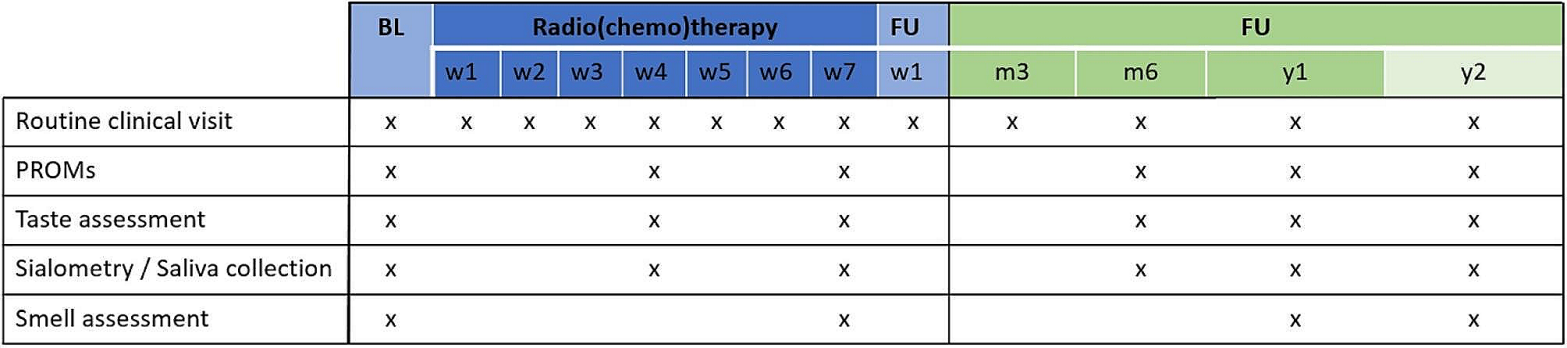
Schematic overview of visits and assessments of patients included in the TASTE study, during and after completion of radio(chemo)therapy. Abbreviations: BL: baseline, FU: Follow-up, PROMs: Patient-reported outcome measures, w: week, m: month, y: year

The TASTE study will be conducted as a prospective, observational multicenter study at the Cantonal Hospital Aarau, the University Hospital Bern and the University Hospital Zurich, Switzerland. As part of this explorative, hypothesis-generating study participants will undergo repeated assessments of their taste and salivary function at time points before, during and after radiation therapy (see Fig. 1). Assessments will include patient-reported outcome measures (PROMs), as well as measurements of taste and smell function and saliva analysis. Participants enter the study prior to starting radiation therapy and will be followed up for two years post-treatment. Participation in the study will not, in any form, influence the treatment received by the patients.

### Study population

We aim to recruit 150 head and neck cancer patients referred to the participating centers for radiation therapy with curative intent, both definitive and post-operative. Those patients are eligible to participate in this study if they are (1) at the minimum 18 years of age, (2) have a Karnofsky performance index of at least 50%, and if (3) the anticipated mean radiation dose to the taste bud bearing tongue mucosa or at least one of the major salivary glands (parotid, submandibular, and/or sublingual gland) is at the minimum 5 Gy.

Exclusion criteria include: (1) pregnancy, (2) pre-existing subjective complete loss of taste at start of radiation therapy, (3) planning CT and radiation therapy with oral stent or spacer, (4) patients with partial or total glossectomy which impairs taste assessments of at least one side of the tongue or impairs contouring of the taste bud bearing tongue mucosa in the planning CT, (5) inability to follow instructions related to study procedures or inability to fill in the questionnaires, (6) inability to provide informed consent. Patients with malignancies of the skin in the head and neck region, sarcomas, solid hematologic tumors and malignancies of the thyroid gland are currently not included in this study.

### Recruitment and screening

Patients are recruited from the departments of Radiation Oncology at the Cantonal Hospital Aarau, the University Hospital Bern and the University Hospital Zurich. Investigators will proactively screen all head and neck cancer patients referred with curative intent for their eligibility. Eligible patients will be informed about the nature of the study, as well as the duration and the procedures involved, during their first consultation by the investigators of the study in the respective centers. If interested, they will receive a detailed study information sheet and a consent form. After ample time for consideration, at the minimum after 24 h, study nurses will phone the patients to confirm their continued interest in participation and schedule their first visit for baseline testing. Signed and dated consent will be obtained before the participant is submitted to any study procedure. No financial compensation will be offered to study participants.

### Sample size

The TASTE study is primarily designed as an explorative, hypothesis-generating study. As there is no formal hypothesis testing, no formal sample size calculation has been performed. We aim to recruit 150 head and neck cancer patients referred for radiation therapy with curative intent. To our knowledge, this constitutes a larger sample size than any other study published so far that investigated the dosimetric impact on taste using the taste bud bearing tongue mucosa and major salivary glands as OAR structures [17] or used semi-objective taste testing in addition to PROMs [11, 14]. Furthermore, this will represent the largest cohort to date with analysis of radiation-associated saliva changes. Thus far, studies investigating changes in saliva composition after radiotherapy to the head and neck region included a maximum of 138 patients [21].

### Outcomes

The primary endpoint is defined as the patient-reported taste impairment assessed by the MDASI-HN (MD Anderson Symptom Inventory Head and Neck module) questionnaire 12 months post-treatment. By choosing this time point we will be able to assess late radiation-associated taste impairment.

Secondary endpoints are taste impairment diagnosed using taste strips at 12 months and 2 years post-treatment, as well as patient-reported taste impairment 2 years post-treatment.

### Assessment of outcomes

Throughout this study participants will undergo repetitive (semi-)objective and subjective assessments of their taste and salivary function. Assessments will include PROMs, as well as measurements of taste and smell function and saliva analysis. Taste assessments, olfactory testing, and saliva collection will be performed at the respective Department of Radiation Oncology by a study nurse or clinical staff. Quality of life questionnaires will be distributed to the patients by a study nurse, secretary, or physician.

Baseline assessments should ideally be conducted prior to starting radiation therapy. However, it can be performed within the first week of radiotherapy at the latest. It should not be performed earlier than two months before the start of treatment. We generally aim to schedule the baseline assessment during the patient visit for the treatment planning computed tomography (CT).

Assessments during treatment will be done in the 4th week of radiation therapy (+/- 1 week) and in the last week of treatment (+/- 1 week). Follow-up assessments at 6 months post-therapy can be performed within a timeframe of 5–7 months after the end of therapy, and the assessments at 1 and 2 years within +/- 3 months. At the minimum, participants are expected to complete the assessments at baseline and 1 year after the end of radiation therapy. Assessments at all other time points are strongly encouraged but will be optional. Olfactory assessment will only be performed at baseline, end of radiotherapy, and during follow-up assessment 1 and 2 years post-treatment. Figure 1 shows a schematic overview of all planned visits and assessments of head and neck cancer patients enrolled in the TASTE study.

During the study visits, assessments will be performed in the following order:

1. Questionnaires (at least MDASI-HN questions 8 (appetite), 10 (dry mouth), and 20 (taste) are to be completed before the visit)
2. Collection of unstimulated saliva
3. Smell assessment
4. Taste assessment (lateralized testing)
5. Collection of stimulated saliva

#### Questionnaires

Throughout the study various patient-reported outcome measures (PROMs) will be collected at different time points. Participants will be asked to complete two separate quality of life questionnaires: (1) the MDASI-HN, a questionnaire developed to assess symptom burden in head and neck cancer patients, and (2) a taste and smell-specific questionnaire developed for this study. All questionnaires are currently performed in paper-and-pen format. An electronic database is currently being established.

The MDASI-HN is a validated head and neck cancer-specific questionnaire that includes MDASI’s 13 core symptoms common across all cancers and nine symptoms relevant to head and neck cancers, as well as six interference items that assess how symptoms interfere with daily life [22]. Patients are asked to grade the presence and severity of symptoms in the last 24 h on an 11-point Likert scale with 0 meaning not present and 10 being the most severe.

Due to the lack of an appropriate taste and smell-specific questionnaire, we created one for use in the TASTE study which includes selected questions from both the Vanderbilt Head and Neck Cancer Symptom Survey (version 2.0, questions 33–36) [23] and the Modified Monell-Jefferson Taste & Smell Questionnaire (questions 1, 26, 33, 37, 41, 45). In four parts this questionnaire aims to get a more detailed view of the subjective changes to taste and smell patients experience during and after radiotherapy. Patients are asked to rate changes (normal, diminished, absent, distorted, heightened) in the different taste qualities (sweet, salty, sour, bitter) and report any phantom oral sensations. In addition, the questionnaire assesses how changes in taste and smell, if present, affect appetite and food intake.

Participants will be asked to complete the questionnaires prior to taste and smell testing. For all follow-up visits after completion of radiation therapy the questionnaires will be distributed to the patients prior to the appointment. They are asked to complete these at home and bring them back on the day of the assessment sessions. Quality of life questionnaires will be distributed at baseline, during week 4 and the last week of radiotherapy, and at 6 months, 1 year and 2 years post-therapy.

#### Smell assessment

Smell assessment is performed using 12 commercially available scent pens representing everyday odors (“ODOFIN Sniffin’ Sticks”, Burghart, Wedel, Germany). The Sniffin’ Sticks test is validated in many countries and allows semi-objective assessment of the olfactory performance of patients [24].

Smell assessment is carried out in a properly ventilated odorless room. Participants are asked not to eat, drink or smoke for at least 60 minutes prior to testing. The test consists of 12 felt-tip pens with different familiar odors. For each pen four answer options are presented to the participant on a multiple-choice card. For odor presentation, the cap is removed from the pen and the tip positioned approximately 2 cm in front of the nostrils for about 3 seconds, according to the manufacturer’s instructions. The patient is asked to inhale the odor and identify it using the multiple-choice card. The Sniffin’ Sticks test is a forced-choice test. When a patient is unable to identify an odor, they are encouraged to guess. Correctly identified odors equal 1 point, thus the maximum score is 12. For analysis, the total score is calculated and not identified odors are documented. Total testing time is approximately 10 min. We aim to perform olfactory assessment at baseline, end of radiation therapy, 1 year post-therapy and 2 years post-therapy.

#### Taste assessment

Taste assessment is performed using commercially available, validated filter paper taste strips (“Taste Strips”, Burghart, Wedel, Germany) with increasing concentrations of sweet, sour, salty and bitter tastants to specifically analyze for impairment of the different taste qualities [25]. Taste is assessed for both the left and right side of the tongue separately. In detail, the taste strips are impregnated with the following tastants and concentrations: (1) sweet: sucrose (0.05 g/ml, 0.1 g/ml, 0.2 g/ml, 0.4 g/ml); (2) sour: citric acid (0.05 g/ml, 0.09 g/ml, 0.165 g/ml, 0.3 g/ml); (3) salty: sodium chloride (0.016 g/ml, 0.04 g/ml, 0.1 g/ml, 0.25 g/ml); (4) bitter: quinine hydrochloride (0.0004 g/ml, 0.0009 g/ml, 0.0024 g/ml, 0.006 g/ml). The test does not assess umami taste.

**Fig. 2:**
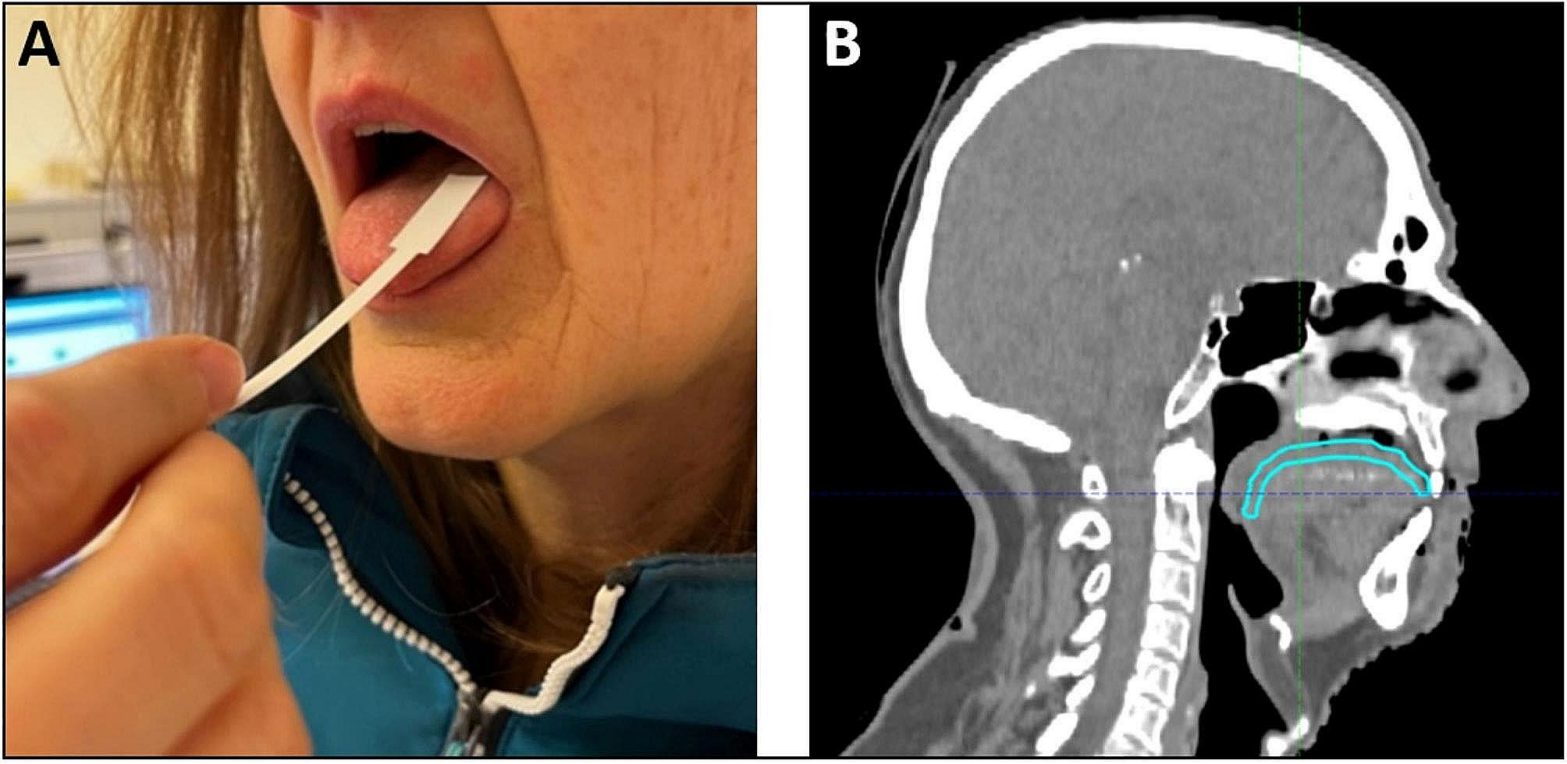
**A:** Lateralized taste assessment. Taste strips are separately placed left and right to the midline of the extended tongue. **B:** Example of contouring of the taste bud bearing tongue mucosa (cyan) as performed in this study according to Stieb et al., 2021 [16]

Participants are asked not to eat, drink or smoke for 1 h prior to testing. Testing is commenced with the neutral control taste strip to let patients familiarize themselves with the taste of the bland taste strip. The taste strips are then placed on the right or left side of the midline of the extended tongue in a pseudo-randomized manner in increasing concentration (according to the manufacturer’s instructions) and moved across the surface (Fig. 2A). Testing always starts on the right side of the tongue, afterwards sides are alternated. The order of tastants applied varies between the left and right side of the tongue. After each taste strip participants are asked to identify the taste and to rinse their mouth with a small amount of plain water. Each correctly identified strip scores one point, hence a maximum of 16 points can be achieved per test if all tastants are correctly identified. Two control strips without tastants are included per tongue side in the testing order, but not counted towards the final score. Therefore, contrary to smell testing this is not a forced choice test, ‘no taste detected’ is an acceptable answer (score 0). A total taste score is calculated for each patient (0–16). In addition, a specific taste score is calculated for each taste quality (0–4). Total testing time is approximately 20 min. We aim to perform taste testing at baseline, during week 4 and the last week of radiotherapy, and at 6 months, 1 year and 2 years post-therapy.

#### Saliva analysis

At each assessment time point two saliva samples are collected: unstimulated and stimulated saliva. The objective of the whole saliva sialometry test is to measure the volume of unstimulated and stimulated saliva in order to objectively assess salivary gland function. Patients are instructed to abstain from eating (including chewing gum), drinking, smoking and oral hygiene for at least one hour before collection.

First, unstimulated saliva is collected into a test tube over a 5 min time period. Subsequently, stimulated saliva will be collected over a 10 min time period while the participant is chewing on a piece of inert paraffin wax (Parafilm M, Sigma). Directly after each collection, the volume of saliva produced will be documented. In addition, the pH value of the saliva will be measured immediately using a calibrated pH meter (Five Go with LE 422, Mettler Toledo). Afterwards, all saliva samples will be centrifuged for 2 min at 1920 x g to remove debris. The supernatant is collected, aliquoted and then stored at -80 °C until further processing. Until freezing, the saliva samples are kept at 6 °C using cooling racks and cold packs. Analysis of the saliva samples (electrolyte composition, proteins, amylase) will later be performed at the Institute for Laboratory Medicine, Cantonal Hospital Aarau.

To ensure reproducibility, we aim to collect saliva samples at the same time of day (+/- 1 h) for each visit, as diurnal changes have been described in saliva previously [26–28]. Total time needed for saliva collection (unstimulated and stimulated) is approximately 20 min. We aim to collect saliva at baseline, during week 4 and the last week of radiotherapy, and at 6 months, 1 year and 2 years post-therapy.

#### Collection of clinical data

Treatment-related information is recorded for each participant. Clinical parameters, such as tumor characteristics, performance status and weight are obtained from the medical chart. Before each taste and smell assessment, patients will be specifically asked about past COVID-19 infections.

In detail, the following information will be collected for each participant in order to evaluate the potential effect of each factor on the outcomes listed above:

- Age, sex
- Tumor localization, tumor stage and nodal stage
- Laterality of the radiation therapy (unilateral vs. bilateral)
- Radiation dose to the taste bud bearing tongue mucosa (Fig. 2B) and salivary glands
- Chemotherapy and/or targeted therapy
- Tumor control post-therapy
- Major oral surgery
- Smoking status and/or alcohol consumption
- Relevant comorbidities (e.g. Sjögren’s syndrome, Parkinson’s disease, COVID-19)
- Medication

Throughout radiation therapy routine clinical visits are conducted weekly. Patients will be seen regularly until acute high-grade toxicities improve. Follow-up clinical visits for all head and neck cancer patients are scheduled at 3 months, 6 months, 1 year and 2 years after completion of treatment in the respective Department of Radiation Oncology. Toxicity is systematically assessed during those visits using the CTCAE v5.0 scale.

### Statistical methods

The TASTE study is an explorative, hypothesis-generating study. Hence, there will be no formal hypothesis testing. Statistical analysis will be performed using a recognized statistical package such as SPSS. A significance level of α = 0.05 (two-sided) will be used and statistical adjustments for multiple testing will be considered. Descriptive statistics will be applied for description of the study cohort, the results of the (semi-) objective and subjective study assessments, and the dose to the taste bud bearing tongue mucosa and the salivary glands. Univariable, binary logistic regression analysis will be performed to test for a significant correlation of clinical and dosimetric variables, smell assessment, saliva output and composition with the primary endpoint (subjective taste impairment at 12 months post therapy). Multivariable NTCP model development will be performed with stepwise forward selection, based on likelihood-ratio, to select the most important predictor(s).

### Timeline

The TASTE study is designed so that assessment time points coincide with routine clinical follow up visits for all head and neck cancer patients. Hence, generally no additional visits will be necessary for the patients. In addition, the assessments are non-invasive. Due to the low additional effort for patients, we estimate an accrual rate of 50% at the Cantonal Hospital Aarau. Data from the first five months of recruitment indicate this to be a realistic figure. Accrual rates for the other two participating centers are expected to be lower (around 20%), due to competing studies involving head and neck cancer patients. We estimate that recruitment will be completed two years after opening the study to multicenter.

## Discussion

Taste impairment is a frequent side effect of radiotherapy to the head and neck area causing significant morbidity and negatively affecting QoL. Yet, there is very limited research thus far. To our knowledge, the TASTE study is the most comprehensive study evaluating taste impairment in head and neck cancer patients undergoing radiation therapy.

Few studies to date have analyzed the impact of the radiation dose on taste impairment, many with major limitations. So far, only one study evaluated the dose toxicity relationship for taste impairment using the taste bud bearing tongue mucosa as OAR structure [17]. However, no baseline values for taste impairment were available for the patients included. This represents a significant limitation, since around 10% of head and neck cancer patients report moderate-to-severe taste impairment already before treatment [29]. In most studies, only the correlation between taste impairment and the mean, median, minimum or maximum dose to the structures have been evaluated [11–14, 17]. No systematic analysis of dose-volume-histogram data has been performed. Furthermore, of the existing studies many include patients in their study cohort treated with 3D conformal radiotherapy which applies a uniform dose to the tongue or large parts of it [9–11, 30]. This technique does not reflect the current state-of-the-art intensity-modulated radiotherapy (IMRT), which allows the dose to be distributed with high conformality around the tumor with a low dose bath around it.

Taste impairment has been shown to be significantly correlated to patient-reported xerostomia using QoL questionnaires [11]. A significant correlation between the mean dose to the parotid glands and taste impairment has been postulated [12, 13, 17], but to date the data remains conflicting. Chen et al. showed that the mean radiation dose to one or both parotid glands predicts taste impairment [12]. In contrast, Fernando et al. found no correlation between the volume of the irradiated parotid glands (bilateral) and taste impairment and concluded that irradiation of the tongue is the primary causative factor of dysgeusia rather than radiation-induced xerostomia [9]. Furthermore, different patterns have been shown between taste impairment and salivary dysfunction, with the latter often being more long-lasting [31]. It should be noted that studies analyzing the correlation between taste impairment and xerostomia thus far used a variety of different assessment methods which limits comparability. Overall, taste-specific studies correlating taste with sialometry are rare, often with small patient cohorts [14], and most do not consider the dose received by the salivary glands as a variable. The TASTE study aims to fill this gap.

Information on how radiation-associated changes in saliva composition and pH changes affect taste is also lacking to date. Decreased salivary pH [21, 32, 33], as well as dynamic changes in electrolyte composition [33] and salivary proteins such as amylase [34] during and after radiotherapy have previously been demonstrated [35]. As salivary constituents interact with tastants and taste receptors, they play an important role in taste sensitivity. Salty taste is dependent on the background levels of sodium chloride that taste receptors are adapted to. Changes in pH, and thus free hydrogen ions, will alter sour taste. Amylase levels might affect perceived sweetness. Bitter taste has been associated with proline-rich proteins [36].

The TASTE study will provide information on late radiation-associated taste impairment taking into account dose-volume histogram (DVH) data of the taste bud bearing tongue mucosa and salivary glands, baseline taste status, and baseline salivary gland function. Patients enter the study prior to commencing radiation therapy and will be followed up for 2 years post-treatment. Study participants will undergo repetitive (semi-) objective and subjective assessment of their taste and salivary function. Using both patient-reported outcome measures, as well as validated taste testing and saliva measurements, will allow us to better assess radiation-associated toxicities in the context of taste impairment. Discrepancies between patient-reported taste perception and actual measured taste loss have been reported, indicating a potential adaptation to the sensory loss [14, 37]. In a study by Sapir et al. only patient-reported xerostomia, but not salivary output, was significantly correlated with taste impairment [11]. Smell assessment was included in the TASTE study as the sense of smell and taste are closely interlinked and loss of smell can disrupt the sense of taste if treatment involves olfactory bulbs or nasal cavity.

We also aim to evaluate whether radiation-associated taste impairment mid- or end of treatment predicts for late taste impairment. Taste impairment generally occurs by week three to four of radiotherapy [9, 15, 38], with some studies reporting maximum taste dysfunction during radiation therapy at around 40 to 60 Gy (Gy) [30, 39]. However, conflicting data exists on the recovery of taste sensation over time. Some studies demonstrate rapid recovery following radiotherapy with taste thresholds returning to baseline by one to six months post-treatment [10, 14, 30]. In other studies, a significant proportion of patients report long-term taste impairment even several years later [2, 17, 38]. While taste appears to markedly improve in the first year after radiotherapy, gradual improvement can continue years after treatment and appears to plateau after five years [40]. In general, data on late taste impairment and the time-dependent course of recovery is limited as most studies only documented short-term follow-up. Patients included in this study will be followed up for 2 years post treatment.

In addition to taste impairment in general, we will evaluate the effects of the radiation dose to the taste bud bearing tongue mucosa on different taste qualities. The specific qualities of taste are known to result from activation of different types of taste cells via distinct receptors and signaling pathways [6]. Therefore, radiation might affect taste qualities differently depending on the underlying pathomechanism. Besides validated taste assessment with taste strips, the participants are asked to complete a taste-specific questionnaire focusing on changes in the different taste qualities. Few studies have analyzed the effect of radiotherapy on individual taste qualities, with often conflicting results. Some studies reported that bitter and salty taste qualities were affected the earliest and more severely in head and neck cancer patients undergoing radiotherapy [31, 38]. Only sour taste was significantly impaired after radiotherapy in another study, while bitter, salty, and sweet tastes were not [15]. Sweet taste appeared to be the most robust in those studies. However, Asif et al. found that sweet and salty taste decline significantly, whereas they only found a trend with bitter, and no change with sour [14]. Yamashita et al. reported an equal impairment pattern of all four basic tastes, as well as umami [39], while the impairment pattern of umami significantly differed from that of the other four taste qualities in another study [41].

Ultimately, the objective of the TASTE study is to develop a NTCP model for late radiation-associated taste impairment. We intend to define dose constraints for the taste bud bearing tongue mucosa and salivary glands. The results of the study may then be further used to evaluate taste-preserving radiotherapy for patients with head and neck cancer, which could significantly reduce the long-term burden in this patient cohort.

### Study status

Open and currently accruing. Recruitment at Cantonal Hospital Aarau commenced in November 2022. Multicenter is projected to open in June 2024. Recruitment is estimated to be completed by June 2026.

## Data Availability

No datasets were generated or analyzed during the current study.

## Abbreviations

TASTE: Therapy-Associated Saliva and Taste change Evaluation
QoL: Quality of life
Gy: Gray
OAR: Organ at risk
NTCP: Normal tissue complication probability
DVH: Dose-volume histogram
PROMs: Patient-reported outcome measures
BL: Baseline
FU: Follow-up
w: Week
m: Month
y: Year
CT: Computed tomography
IMRT: Intensity-modulated radiotherapy
CRF: Case Report Form
